# Association between alcohol consumption and risk of stroke among adults: results from a prospective cohort study in Chongqing, China

**DOI:** 10.1186/s12889-023-16361-9

**Published:** 2023-08-22

**Authors:** Xin Liu, Xianbin Ding, Fan Zhang, Liling Chen, Qinwen Luo, Meng Xiao, Xiang Liu, Yunyun Wu, Wenge Tang, Jingfu Qiu, Xiaojun Tang

**Affiliations:** 1 School of Public Health, Research Center for Medical and Social Development, Chongqing Medical University, Chongqing, China; 2 Institute of Chronic Non-Communicable Disease Control and Prevention, Chongqing Center for Disease Control and Prevention, Chongqing, China; 3 Department of Epidemiology and Health Statistics, West China School of Public Health, Sichuan University, Chengdu, China

**Keywords:** Stroke, Alcohol consumption, Prospective cohort study, Drinking patterns, Interactions, Adults

## Abstract

**Background:**

The incidence of stroke in China is increasing, along with a clear trend in the prevalence of risk factors. Alcohol consumption is also a risk factor for stroke. Many cohort studies have explored the relationship between alcohol consumption and stroke risk. However, findings have been inconsistent.

**Methods:**

We used cluster sampling to select 13 districts and counties (at the same level) in Chongqing, China. Then, we used stratified random sampling to distribute the number of people in each district and county. 23,308 adults aged 30–79 were recruited between October 2018 and February 2019. Follow-up was conducted through a monitoring system and questionnaires until September 2022. Information on alcohol consumption and other covariates was collected using a standardized questionnaire. Participants were asked to report their weekly frequency of drinking over the past year and weekly intake of various alcoholic beverages in general. The frequency of drinking was divided into three categories: 1–2 d/week, 3–5 d/week, and 6–7 d/week. The average daily alcohol consumption is calculated based on the amount of alcohol contained in different alcoholic beverages. It is classified as nondrinker (0 g/day), light (0 to 12 g/day), moderate (13 to 36 g/day), and high (> 36 g/day). Cox proportional hazard regression models were used to estimate the association between alcohol consumption and stroke risk. Results are shown as multivariate-adjusted hazard ratios (HRs) and 95% confidence intervals (95% CIs).

**Results:**

With an average follow-up of 3.80 years, there were 310 new stroke events. The incidence of total stroke was 368.69 per 100,000 person-years. Overall, after adjusting for covariates, moderate alcohol consumption (average daily alcohol consumption 13–36 g/d) was associated with a lower risk of total stroke (HR: 0.48; 95% CI: 0.25–0.92) compared with nondrinkers. The adjusted HR and 95% CI for total stroke and ischemic stroke for those who drank alcohol 6–7 days per week were 0.60(0.37, 0.96) and 0.53(0.30, 0.94), respectively. The risk of total stroke (HR: 0.39; 95% CI: 0.17–0.89) was reduced in a pattern of drinking 6–7 days per week but with a mean alcohol consumption of less than 36 g/d. There was no significant association between alcohol consumption and hemorrhagic stroke.

**Conclusion:**

This study suggests moderate alcohol consumption is associated with a lower risk of total stroke. And healthy drinking patterns should be of more significant concern.

**Supplementary Information:**

The online version contains supplementary material available at 10.1186/s12889-023-16361-9.

## Background

According to the Global Burden of Disease Study (GBD), the lifetime risk of stroke in China is the highest globally, as high as 39.3% [1]. According to the data from China Stroke Prevention and Treatment Report 2019 [2], the incidence of stroke in China has increased in the past 30 years with the development of society and economy. Alcohol consumption is a significant risk factor for stroke, a modifiable lifestyle [3]. A recent review of meta-analyses based on prospective cohort studies has shown that low alcohol consumption strongly diminished the risk of total stroke, ischemic stroke, and hemorrhagic stroke. In contrast, moderate alcohol consumption only reduced the risk of ischemic stroke. In contrast, heavy alcohol consumption was associated with an increased risk of hemorrhagic stroke [4].

However, the relationship between alcohol consumption and stroke is still controversial. Some studies have shown that low-dose alcohol consumption might associate with lower ischemic stroke risk [5–8] and total stroke risk [6, 7] compared to the reference group. Some studies have suggested that light to moderate alcohol consumption could prevent stroke [7, 9, 10]. For example, a previous cohort study among 87,526 female nurses aged 34 to 59 years showed a relative risk of ischemic stroke of 0.3 (95% CI: 0.1–0.7) with 5 to 14 g of alcohol intake per day compared with non-drinkers [11]. However, the findings of some researchers disprove this view. Some studies have concluded that current alcohol consumption is not associated with stroke risk [12, 13]. A cohort study of a middle-aged US population showed that light to moderate alcohol consumption did not significantly reduce the risk of ischemic or hemorrhagic stroke [14]. These controversies may be attributed to heterogeneity in stroke subtypes, potential differential effects of gender and race/ethnic groups, and different types and amounts of alcohol consumption. Many cohort studies and meta-analyses have reached consistent conclusions regarding the relationship between heavy drinking and stroke, suggesting heavy drinking could be a risk factor for stroke development [5, 6, 15, 16].

Chinese drinking culture differs from Western countries, and the type, ethanol content, amount, and mode of alcoholic beverages may vary between ethnic groups [17–20]. Domestic studies focus on the distribution of alcohol consumption amount and type in the population, and these studies are mainly cross-sectional surveys or retrospective studies [21–24]. For example, a cross-sectional study found that light to moderate alcohol consumption was associated with reduced risk of stroke of all types (OR: 0.91; 95% CI: 0.85–0.97) and of ischemic stroke (OR: 0.90; 95% CI: 0.84–0.97) [21]. However, Li et al. Found no significant association between moderate alcohol consumption and ischemic stroke (OR: 0.65; 95% CI: 0.37–1.16) [22]. A review suggested that few studies have focused on drinking patterns, which may play an essential role in alcohol-induced chronic diseases [25]. Cross-sectional studies, however, cannot prove causality. Based on a large sample prospective cohort study, our study can address this limitation. In addition to discussing the amount and frequency of alcohol consumption separately, we further explored the influence of different drinking patterns on the risk of stroke and stroke subtypes and performed detailed subgroup analyses.

This study aimed to explore the effects of different drinking characteristics and patterns on stroke risk using the Han cohort aged 30–79 established by the China Multi-Ethnic Cohort (CMEC) study in Chongqing to provide a reference for formulating public health recommendations on alcohol consumption.

## Materials and methods

### Study population

**Fig. 1:**
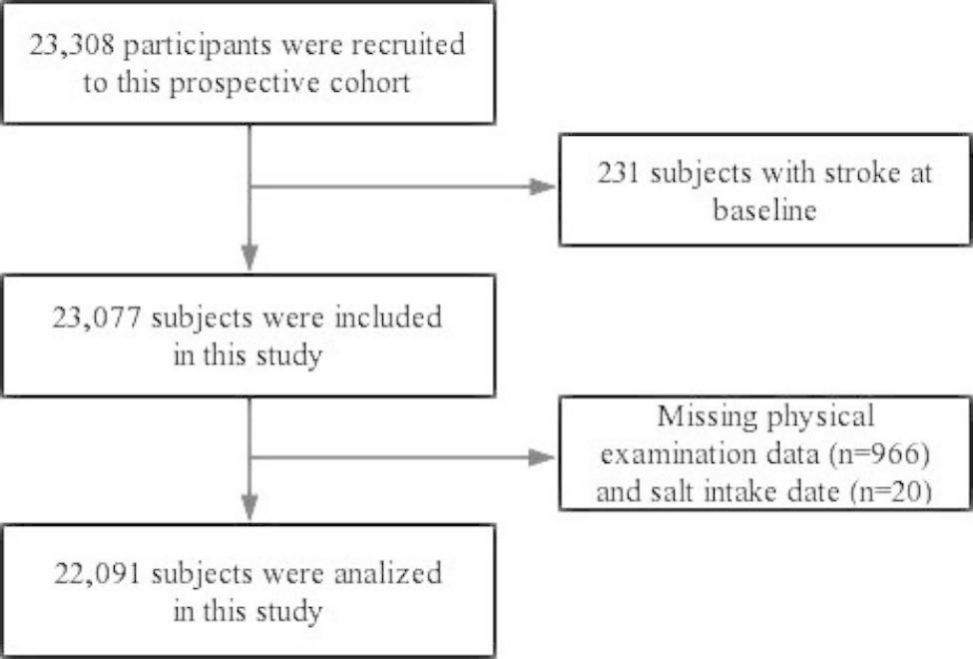
Flowchart for participants’ selection

This is an ongoing community-based prospective study from Chongqing Municipality in southwest China, based on the China Multi-Ethnic Cohort (CMEC) study. Details of the CMEC study design have been described elsewhere [26]. 23,308 Han Chinese participants, aged 30–79 years, who had lived in the local area for half a year or more, were recruited by multi-stage, stratified cluster sampling between September 2018 and February 2019. In brief, the cluster sampling method is adopted to select 13 districts and counties (districts and counties of the peer grade) in Chongqing. Then, according to the age and gender structure of Chongqing in 2018, the number of people in each district and county was allocated by stratified random sampling. The baseline survey included a questionnaire, physical examination, and biological sample collection. Details of the participant selection are shown in Fig. 1. In this study, 22,091 participants were included in the final analysis. The inclusion criteria were: [1] completed the baseline (n = 23,308); [2] without stroke diagnosed by a physician at registration (n = 23,077). The exclusion criteria were: [1] missing data for physical examination (n = 966); [2] missing data for salt intake (n = 20). Follow-up began at the time of the baseline survey and ended in September 2022, with a mean follow-up of 3.8 years. Ethical approval was obtained from the Medical Ethics Review Committee of Sichuan University (K2016038). All participants signed written informed consent.

### Assessment of alcohol consumption

Information about alcohol consumption was obtained by uniformly trained investigators using face-to-face interviews and a unique electronic questionnaire. The survey unit for beer is bottle/week, and 500ml of beer per bottle is the standard. The survey unit for rice wine, wine, high-liquor, and low-liquor is 50 g/week. Our investigators are professionally trained, and each investigator was equipped with a 200ml disposable paper cup, mineral water bottle lid, standard bowl (3.3 inches), and a 10 cm steel ruler to help survey subjects better estimate the volume and weight. Participants were asked, “On average, how many days per week have you drunk alcohol in the past year?” with the following three response categories: “1-2d/week, 3-5d/week, and 6-7d/week.” Participants were also asked about the type of alcohol (beer, rice wine, wine, high-alcohol liquor, low-alcohol liquor) and weekly intake of various kinds of alcoholic beverages in general. The alcohol by volume (ABV) across multiple beverage types was estimated to be 4% (beer), 18% (rice wine), 10% (wine), 52% (high-alcohol liquor), and 38% (low-alcohol liquor) [27, 28]. Alcohol intake is calculated according to the number of grams of alcohol converted from the amount of alcohol contained in different drinks, alcohol intake (g) = alcohol consumption (mL) × alcohol by volume (%)× alcohol mass fraction (g/mL), and alcohol mass fraction is calculated as 0.8 g/mL. The average daily alcohol consumption is obtained by dividing the weekly alcohol consumption by 7. Participants were divided into four groups based on average daily alcohol consumption (g/day) at baseline: nondrinker (0 g/day), light (0 to 12 g/day), moderate (13 to 36 g/day), and high (> 36 g/day) [12, 29].

### Assessment of covariates

Trained researchers conducted face-to-face interviews using structured questionnaires to collect information on sociodemographic characteristics (age, sex, marital status, annual household income, education level), lifestyle factors (smoking status, physical activity), dietary habits (red meat intake, fruits, and vegetable intake, spicy food intake) and disease history (hypertension, diabetes). Participants who reported not smoking at baseline and had smoked less than 100 cigarettes in their lifetime were defined as nonsmokers. Current smokers were defined as those who had smoked more than 100 cigarettes in their lifetime and had smoking behavior within the past six months. Ex-smokers were defined as quitting smoking for over six months and beyond. Those who quit smoking for less than six months are still current smokers [26]. Participants were asked, “During the past year, how often did you participate in physical activity in your spare time?” with the following five response categories: “never or rarely, 1–3 times/month, 1–2 times/week, 3–5 times/week, 6–7 times/week.” We assessed participants’ consumption of red meat, fruits, and vegetables over the past year. According to the World Cancer Research Fund criteria [30], this study defined a cumulative intake of ≥ 100 g/day of pork, beef, and lamb as an excessive intake of red meat. According to WHO recommendations, insufficient intake of fruits and vegetables is defined as less than 400 g of vegetables and fruits per capita day [31]. Regarding spicy food intake, participants were asked, “How frequently did you have spicy foods during the past month?” “Never,” “<1 day/week”, “1–2 days/week”, “3–5 days/week,” or “6–7 days/week.” We defined those who answered “<1 day/week”, “1–2 days/week”, “3–5 days/week,” or “6–7 days/week” as spicy eaters. Body weight (kg) and standing height (m) were measured by uniformly trained staff using standard protocols and calibration instruments at baseline. BMI (kg/m^2^) was calculated by dividing weight in kilograms by the square of height in meters. Underweight was defined as a BMI < 18.5 kg/m^2^, normal weight as a BMI of 18.5 ≤ BMI<24 kg/m^2^, overweight as a BMI of 24 ≤ BMI<28 kg/m^2^, and obesity as a BMI ≥ 28 kg/m^2^ [32]. In this study, hypertension was defined as having an average measured SBP/DBP ≥ 140/90 mmHg or a self-reported physician diagnosis of hypertension. Define diabetes as an FBG level ≥ 7 mmol/L or a previous self-reported physician diagnosis of diabetes [31]. According to the 2007 edition of the Chinese Guidelines for the Prevention and Treatment of Dyslipidemia in Adults [33], participants with dyslipidemia are those who have any of the following conditions: [1] Serum total cholesterol (TC) ≥ 6.22 mmol/l; [2] Triacylglycerol (TG) ≥ 2.26mmol/l; [3] Low-density lipoprotein cholesterol (LDL-C) ≥ 4.14mmol/l; [4] High-density lipoprotein cholesterol (HDL-C) < 1.04mmol/l.

### Follow-up and determination of stroke

This cohort study was followed up annually to determine the outcome events of the subjects. In the follow-up of this project, new stroke events were obtained mainly by matching the individual’s unique ID number or personal code with the Chongqing death registration system and Chongqing cardiovascular disease reporting system, supplemented by 3 years (2019, 2020, 2022) of telephone follow-up. Both methods reduce the loss of follow-up interactively. After each follow-up, the quality control team randomly selected 5% of the survey objects at each investigation site for repeating telephone follow-ups, with a concordance rate higher than 85%. The failed questionnaires were re-followed.

There was a migration of subjects to another province during follow-up. However, this situation is a small proportion of our study population, accounting for 2.2% (486/22,091), which has little influence on the research results. In addition, the subjects were followed up by telephone or found in the monitoring system. So we can also follow their ending events. Second, we tracked people outside the system through telephone follow-up. In 310 stroke events, data were supplemented by telephone follow-up in 4.5% (14/310) and the system in 95.5% (296/310).

Trained staff, blinded to the baseline information, coded all cases with the 10th revision of the International Classification of Diseases (ICD-10). Hemorrhagic stroke was defined as subarachnoid hemorrhage (I60) and cerebral hemorrhage (I61-I62), ischemic stroke was defined as cerebral infarction (I63), and other or unknown types of stroke (I64). We excluded transient ischemic attack (TIA) and chronic cerebral arteriosclerosis.

### Statistical analysis

The subjects’ general characteristics were described after classifying populations into different groups by alcohol intake (nondrinkers, light, moderate, high). Continuous and categorical variables were expressed as median (interquartile range) and percentages. The Kruskal-Wallis H and Chi-square tests or Fisher’s exact tests were also used, respectively. Follow-up person-years were calculated from the date of cohort enrollment to the date of diagnosis of stroke, death, or September 2022, whichever came first. The multivariate Cox proportional hazards regression models assessed the association between different drinking characteristics or drinking patterns and stroke risk. In this study, we used non-drinkers as the reference. Drinking characteristics included the degree of alcohol intake (light, moderate, high), frequency of alcohol consumption (1–2 days/week, 3–5 days/week, 6–7 days/week), and drinking patterns combining the degree of alcohol intake and frequency of alcohol consumption. Results are shown as covariate-adjusted hazard ratios (HRs) and 95% confidence intervals (95% CIs). Proportional hazards assumptions were not violated when assessed using the Schoenfeld residuals (p > 0.05). The selection process of the potential confounders was based on an extensive literature search and then tested by a univariate Cox regression analysis. In this study, we applied two multivariate hazard models. Model 1 was a crude model without any adjustments; model 2 adjusted for gender, age, education level, marital status, smoking status, physical exercise, excessive intake of red meat, insufficient intake of vegetables and fruits, intake of spicy food, BMI, hypertension, diabetes, dyslipidemia, and drinking quantity*gender. The results of the fully adjusted Model 2 are considered final. Considering that the number of men drinkers is higher than that of women drinkers, we additionally analyzed the relationship between alcohol consumption and stroke risk in men.

We used Cox proportional hazard model to plot the adjusted survival curve. Specifically, the dependent variable was whether the participants had a stroke during the follow-up period, and the independent variables were the participants’ degree and frequency of alcohol consumption. The control variables were demographic characteristics, lifestyle factors, and related diseases.

Subgroup analysis was conducted by degree and frequency of alcohol consumption. The study was stratified by different baseline characteristics (including gender, age, education, smoking status, BMI, hypertension, diabetes, and dyslipidemia) to examine potential changes in effects. The HR and 95% CI for stroke were determined by comparing drinkers with non-drinkers. The multiplicative interactions were analyzed using Cox regression. Interaction terms between variables were included in the model. The presence of a multiplicative interaction was determined by the magnitude of the p-value (p<0.05). Sensitivity analyses were performed by excluding participants (n = 3,886) with self-reported malignancy, coronary heart disease, hyperlipidemia, and chronic obstructive pulmonary disease at baseline to verify the stability of our findings.

All statistical analyses were performed using SPSS version 26.0. All statistical tests were two-sided; p < 0.05 was considered statistically significant.

## Results

### Baseline characteristics

At baseline, the average age of the 22,091 participants was 49.56 years. 19,106 (86.49%) participants were defined as nondrinkers, 894 (4.05%) participants as light drinkers, 1,198 (5.42%) participants as moderate drinkers, and 893 (4.04%) participants as high drinkers. Compared with light and moderate drinkers, high drinkers were older, more likely to be current smokers, to have excessive red meat intake, to have a higher proportion of hypertension at baseline, and preferred to have higher FBG and TC levels (P < 0.05) **(**Table 1**)**.

**Table 1:** Baseline characteristics by category of average daily alcohol consumption.

Variables	Total	Degree of alcohol intake				P value
		Nondrinkers	Light	Moderate	High	
N, %	22,091(100.00)	19,106(86.49)	894(4.05)	1,198(5.42)	893(4.04)	
**Age at baseline, years**	49.56	49.16	51.37	51.53	55.30	**<0.001**
**Gender, %**						**<0.001**
Male	46.77	40.13	76.51	92.65	97.65	
Female	53.23	59.87	23.49	7.35	2.35	
**Marital status, %**						0.973
Married/cohabiting	87.93	87.59	89.60	90.48	90.26	
Separated/divorced	6.58	6.65	6.71	6.09	5.60	
Widowed/unmarried	5.49	5.76	3.69	3.42	4.14	
**Annual family income, yuan %**						1.000
< 12,000	11.11	11.23	10.29	9.27	11.87	
12,000–19,999	12.80	13.11	11.41	10.18	11.09	
20,000–59,999	34.42	34.50	34.34	34.14	33.03	
60,000–99,999	21.16	21.09	21.48	23.29	19.48	
≥ 100,000	20.51	20.07	22.48	23.12	24.52	
**Degree of education, %**						0.837
Primary or below	32.51	32.45	32.21	28.96	38.86	
Junior high school	32.27	32.13	33.56	33.56	32.25	
High school or above	35.22	35.42	34.23	37.48	28.89	
**Smoking status, %**						**<0.001**
Non-smoker	73.18	79.74	46.98	28.63	18.81	
Current smoker	20.69	15.30	39.26	56.93	68.65	
Ex-smoker	6.13	4.96	13.76	14.44	12.54	
**Physical exercise, %**						0.994
Never	42.26	41.67	42.06	45.99	50.06	
1–3 times/month	8.26	8.47	8.39	7.26	4.93	
1–2 times/week	11.75	11.92	12.30	11.27	8.06	
3–5 times/week	7.44	7.57	7.16	6.84	5.71	
6–7 times/week	30.30	30.37	30.09	28.63	31.24	
**Insufficient intake of vegetables and fruits, %**						0.841
No	56.31	57.00	53.36	51.00	51.74	
Yes	43.69	43.00	46.64	49.00	48.26	
**Excessive intake of red meat, %**						**0.013**
No	81.51	82.95	78.75	73.37	64.39	
Yes	18.49	17.05	21.25	26.63	35.61	
**Spicy food intake, %**						0.105
No	14.09	15.24	6.71	6.18	7.50	
Yes	85.91	84.76	93.29	93.82	92.50	
**BMI, %**						0.938
Underweight	1.49	1.58	1.01	1.25	0.56	
Normal	42.65	43.69	37.81	34.64	35.95	
Overweight	41.16	40.35	46.20	47.58	44.79	
Obesity	14.69	14.38	14.99	16.53	18.70	
**Hypertension, %**						**0.010**
No	65.17	66.85	59.84	58.01	44.23	
Yes	34.83	33.15	40.16	41.99	55.77	
**Diabetes, %**						0.699
No	90.40	90.73	89.71	88.98	86.00	
Yes	9.60	9.27	10.29	11.02	14.00	
**Dyslipidemia, %**						0.113
No	71.74	72.96	67.79	66.11	57.11	
Yes	28.26	27.04	32.21	33.89	42.89	
**SBP, mmHg**	127.67	127.00	130.00	131.33	136.00	**<0.001**
**DBP, mmHg**	78.00	77.33	80.33	82.33	83.67	**<0.001**
**FBG, mmol/L**	5.26	5.24	5.31	5.40	5.50	**<0.001**
**TC, mmol/L**	4.93	4.91	5.06	5.03	5.17	**<0.001**
**HDL-C, mmol/L**	1.51	1.52	1.48	1.44	1.49	**<0.001**
**LDL-C, mmol/L**	2.68	2.67	2.76	2.72	2.70	**<0.001**
Types of alcoholic beverages*						**<0.001** ^**#**^
Beer	3.30	-	34.00	24.71	14.33	
Rice wine	0.10	-	1.57	0.42	0.22	
Wine	0.24	-	5.82	0.17	0.00	
High-alcohol liquor	8.59	-	44.07	65.53	80.40	
Low-alcohol liquor	1.29	-	14.54	9.18	5.04	

Note: Data displayed as median or frequency (percentage). *Types of alcoholic beverages in drinkers. ^#^Using Fisher’s exact test. Abbreviations: BMI, body mass index; SBP, systolic blood pressure; DBP, diastolic blood pressure; FBG, fasting blood glucose; TC, total cholesterol; HDL-C, high-density lipoprotein cholesterol; LDL-C, low-density lipoprotein cholesterol.

### 2. The relationship between alcohol consumption and stroke risk

After an average of 3.80 years of follow-up (84080.54 person-years), a total of 310 incident stroke cases (approximately 368.69/100,000 person-years) were identified (including 245 ischemic stroke, 59 hemorrhagic stroke, and 6 other stroke cases). In model 2, compared with nondrinkers, moderate drinkers had a significantly lower risk of total stroke (HR: 0.48; 95% CI: 0.25–0.92). High alcohol consumption was not associated with stroke risk. In addition, by defining another standard of alcohol consumption and then analyzing it, the results were consistent (**Supplementary Table 1**). In different drinking frequencies, compared with nondrinkers, the multivariable-adjusted HRs (95% CIs) for total stroke and ischemic stroke in subjects who drank 6–7 days per week was 0.60(0.27, 0.96) and 0.53(0.30, 0.94), respectively **(**Table 2**)**. The risk of total stroke was reduced by 61% (95% CI: 0.17–0.89) among those who drank alcohol 6–7 days per week and had an average alcohol intake of less than 36 g/d **(**Table 3**)**.

**Table 2:** Association between drinking characteristics and stroke risk among 22,091 participants (HRs and 95% CIs).

Drinking characteristics	Incidence Density (1/100,000 Person-Years)	Model 1	Model 2
Total stroke (N = 310)			
Nondrinkers	375.55	1.00	1.00
Degree of alcohol intake			
light	292.11	0.78(0.42,1.47)	0.71(0.36,1.40)
moderate	217.81	0.58(0.31,1.09)	**0.48(0.25,0.92)**
high	504.18	1.34(0.82,2.19)	0.65(0.37,1.12)
Drinking frequency			
1-2d/week	175.37	0.47(0.21,1.05)	0.55(0.24,1.27)
3-5d/week	257.72	0.69(0.34,1.39)	0.64(0.31,1.31)
6-7d/week	473.17	1.26(0.82,1.93)	**0.60(0.37,0.96)**
**Ischemic stroke** **(N = 245)**			
Nondrinkers	299.89	1.00	1.00
Degree of alcohol intake			
light	204.48	0.68(0.32,1.45)	0.71(0.33,1.53)
moderate	196.03	0.65(0.34,1.27)	0.57(0.29,1.13)
high	326.24	1.09(0.60,2.00)	0.57(0.30,1.12)
Drinking frequency			
1-2d/week	146.14	0.49(0.20,1.19)	0.65(0.26,1.60)
3-5d/week	225.51	0.75(0.36,1.60)	0.76(0.35,1.65)
6-7d/week	308.59	1.03(0.61,1.74)	**0.53(0.30,0.94)**
**Hemorrhagic stroke** **(N = 59)**			
Nondrinkers	70.16	1.00	1.00
Degree of alcohol intake			
light	87.63	1.25(0.39,4.01)	0.77(0.18,3.26)
moderate	—	—	—
high	148.29	2.11(0.84,5.29)	0.77(0.26,2.26)
Drinking frequency			
1-2d/week	29.23	0.42(0.06,3.02)	0.30(0.04,2.49)
3-5d/week	32.22	0.46(0.06,3.33)	0.30(0.04,2.27)
6-7d/week	123.44	1.76(0.76,4.10)	0.61(0.22,1.64)

Model 1: unadjusted crude model; Model 2: adjusted for gender, age, education level, marital status, smoking status, physical exercise, excessive intake of red meat, insufficient intake of vegetables and fruits, intake of spicy food, BMI, hypertension, diabetes, dyslipidemia, and drinking quantity*gender.

**Table 3:** Association between drinking patterns and stroke risk in 22,091 participants (HRs and 95% CIs).

Drinking patterns		Incidence Density (1/100,000 Person-Years)	Model 1	Model 2
Total stroke (N = 310)				
Nondrinkers		375.55	1.00	1.00
1-2d/week	0-36 g/d	188.32	0.50(0.22,1.13)	0.65(0.29,1.48)
	>36 g/d	—	—	—
3-5d/week	0-36 g/d	298.80	0.80(0.38,1.69)	0.74(0.34,1.59)
	>36 g/d	131.34	0.35(0.05,2.50)	0.34(0.05,2.45)
6-7d/week	0-36 g/d	281.61	0.75(0.36,1.59)	**0.39(0.17,0.89)**
	>36 g/d	673.64	**1.80(1.08,2.97)**	0.72(0.41,1.26)
**Ischemic stroke** **(N = 245)**				
Nondrinkers		299.89	1.00	1.00
1-2d/week	0-36 g/d	156.93	0.52(0.22,1.27)	0.71(0.29,1.75)
	>36 g/d	—	—	—
3-5d/week	0-36 g/d	256.11	0.86(0.38,1.92)	0.84(0.37,1.94)
	>36 g/d	131.34	0.44(0.06,3.13)	0.45(0.06,3.25)
6-7d/week	0-36 g/d	201.15	0.67(0.28,1.63)	0.42(0.17,1.04)
	>36 g/d	421.03	1.41(0.75,2.65)	0.62(0.31,1.25)
**Hemorrhagic stroke** **(N = 59)**				
Nondrinkers		70.16	1.00	1.00
1-2d/week	0-36 g/d	31.39	0.45(0.06,3.24)	0.52(0.07,3.83)
	>36 g/d	—	—	—
3-5d/week	0-36 g/d	42.69	0.61(0.08,4.41)	0.46(0.06,3.41)
	>36 g/d	—	—	—
6-7d/week	0-36 g/d	40.23	0.57(0.08,4.15)	—
	>36 g/d	210.51	**3.00(1.20,7.51)**	0.86(0.29,2.55)

Model 1: unadjusted crude model; Model 2: adjusted for gender, age, education level, marital status, smoking status, physical exercise, excessive intake of red meat, insufficient intake of vegetables and fruits, intake of spicy food, BMI, hypertension, diabetes, dyslipidemia, and drinking quantity*gender.

Because of the small sample size and few cases among women, we only showed the association between different drinking characteristics and drinking patterns and the risk of stroke incidence among men (Supplementary Table S2 and S3). The results showed that in model 2, compared with nondrinkers, moderate drinking and drinking 6–7 days per week were associated with a reduced risk of total stroke, with HRs (95% CIs) of 0.48 (0.25, 0.92) and 0.58 (0.36, 0.94), respectively. In the pattern of drinking 6–7 days per week with an average alcohol intake of less than 36 g/d, the risk of total stroke was reduced (HR: 0.41; 95% CI: 0.18–0.93).

### 3. Subgroup analysis

**Fig. 2:**
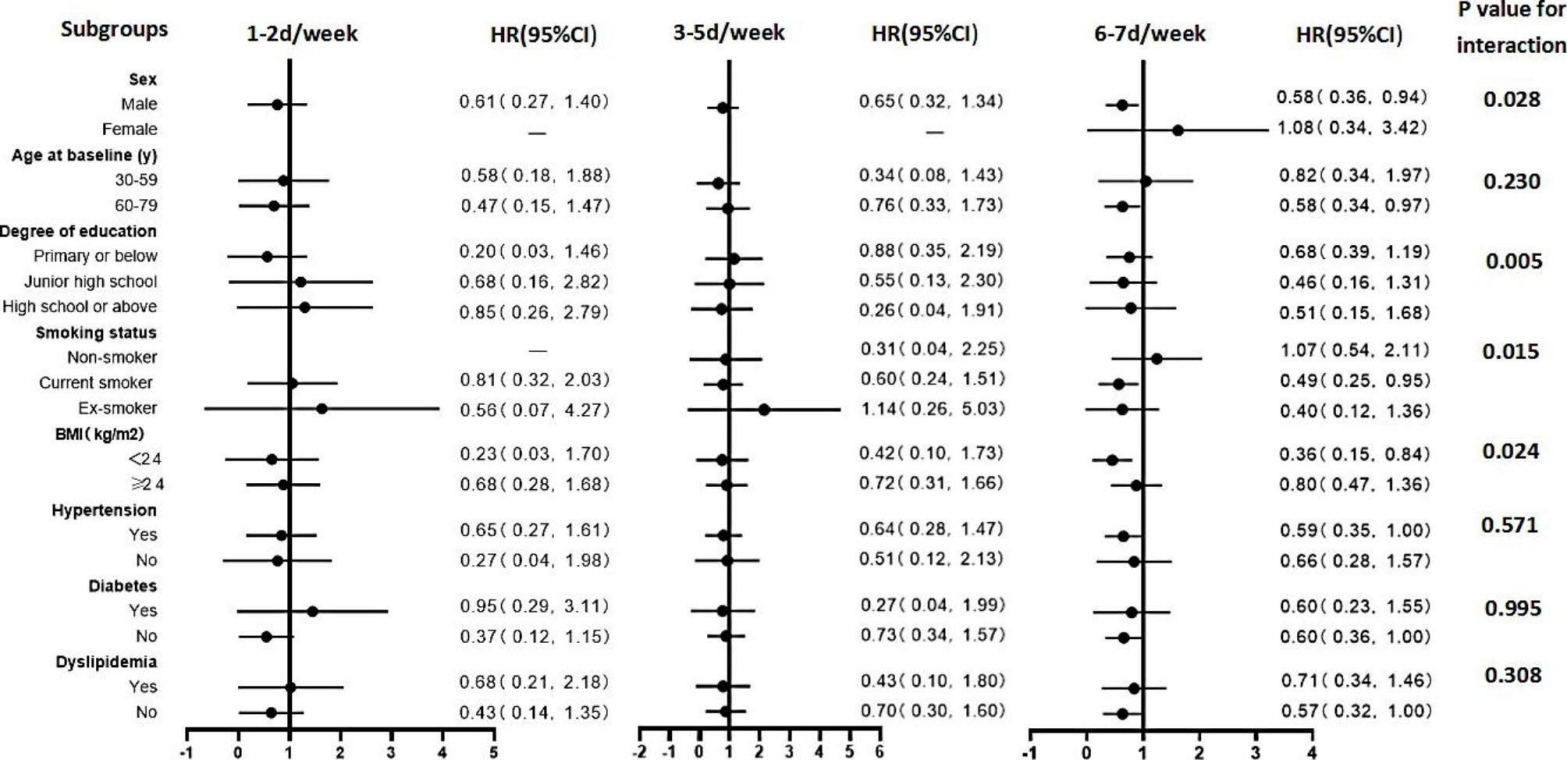
Subgroup analysis based on the association between frequency of drinking and stroke risk. The HRs and 95% CIs for stroke were derived from comparing drinkers versus nondrinkers. Values were obtained from a Cox proportional hazards analysis. The analysis was adjusted according to gender, age, education level, marital status, smoking status, physical exercise, excessive intake of red meat, insufficient intake of vegetables and fruits, spicy food intake, BMI, hypertension, diabetes, and dyslipidemia. Round dots represent the HRs, and horizontal lines represent the corresponding 95% CIs.

**Fig. 3:**
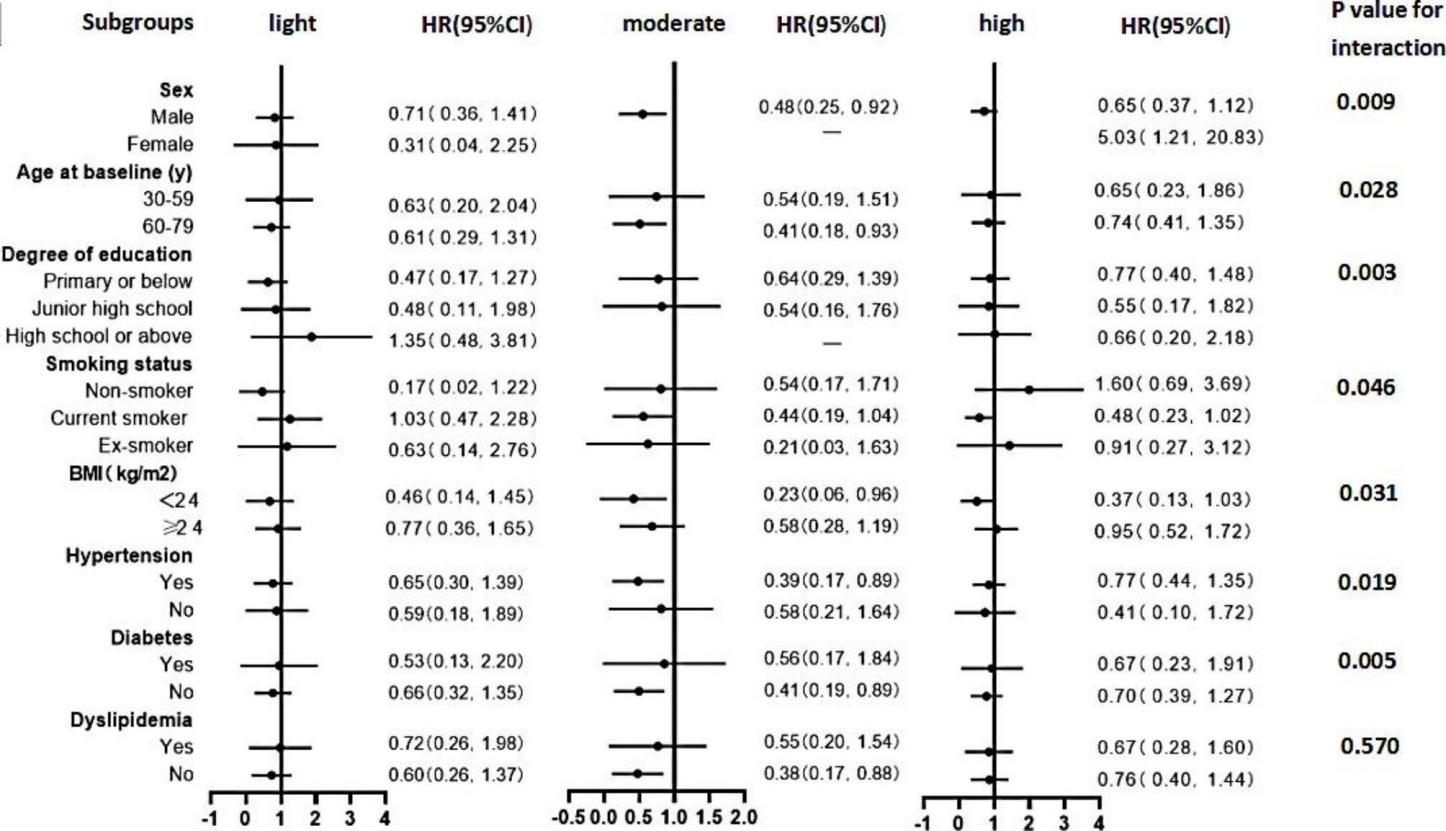
Subgroup analysis based on the association between the degree of alcohol intake and stroke risk. The HRs and 95% CIs for stroke were derived from comparing drinkers versus nondrinkers. Values were obtained from a Cox proportional hazards analysis. The analysis was adjusted according to gender, age, education level, marital status, smoking status, physical exercise, excessive intake of red meat, insufficient intake of vegetables and fruits, spicy food intake, BMI, hypertension, diabetes, and dyslipidemia. Round dots represent the HRs, and horizontal lines represent the corresponding 95% CIs.

We analyzed the relationship between the degree of alcohol intake, frequency of alcohol consumption, and the risk of stroke in specific population subgroups **(**Figs. 2 and 3**)**. HRs and 95% CIs are listed after adjustment for model 4. In addition, some rows in the results were removed because the sample size was too small, there were no cases, or the confidence interval was too large.

In the stratified analysis, the negative association between drinking 6–7 days per week and stroke was more pronounced in men (P _interaction_=0.028), current smokers (P _interaction_=0.015), and those with BMI < 24 kg/m^2^ (P _interaction_=0.024), with HRs (95% CIs) of 0.58 (0.36, 0.94), 0.49 (0.25, 0.95), and 0.36 (0.15, 0.84), respectively**(**Figure 2**)**. Figure 3 shows that the negative association between moderate alcohol consumption and stroke was corroborated in males, 60–79 years, with BMI < 24 kg/m^2^, with hypertension, and without diabetes (P _interaction_<0.05).

### 4. Sensitivity analysis

To assess the robustness of our results, after excluding 3,886 individuals who self-reported having malignancy, coronary heart disease, hyperlipidemia, and chronic obstructive pulmonary disease at baseline in this study, the results showed no significant change in the observed relationship **(Supplementary Tables 4 and 5)**.

## Discussion

This prospective cohort study revealed that alcohol consumption may be associated with a lower stroke risk than non-drinkers. In different drinking characteristics, we found that moderate alcohol consumption (13–36 g/day) was associated with a lower risk of total stroke, and drinking 6 to 7 days per week was associated with a lower risk of total stroke and ischemic stroke. For drinking patterns, a drinking frequency of 6–7 days per week drinking but with an average alcohol intake of less than 36 g/d reduced the risk of total stroke. There was no significant association between alcohol consumption and risk of hemorrhagic stroke.

Current domestic studies have mainly focused on the distribution of alcohol consumption amount and type in the population, with cross-sectional, retrospective studies. Our study prospectively assessed the negative association between alcohol consumption and stroke risk, and the findings were consistent with previous studies (5–11, 34–35). For example, the results of a meta-analysis that included 27 prospective studies showed that low alcohol intake was associated with a reduced risk of total stroke (RR: 0.85; 95% CI: 0.75–0.95; P = 0.005) and ischemic stroke (RR: 0.81; 95% CI: 0.74–0.90; P < 0.001), but no significant effect on hemorrhagic stroke [34]. Another meta-analysis showed a reduced risk of ischemic stroke with moderate alcohol consumption (1–2 drinks/day) (RR: 0.92; 95% CI: 0.87–0.97) [5]. In a study of 83,578 women free of diagnosed cardiovascular disease and cancer at baseline, moderate alcohol consumption (5-14.9 g/d) was associated with a 21% reduction in the risk of total stroke [9]. Similarly, a cohort study from rural Tianjin, China, found that alcohol consumption may be associated with a lower risk of total stroke than nondrinkers (HR: 0.68; 95% CI: 0.54–0.88) [35]. However, the amount or frequency of alcohol consumption alone is insufficient to assess the association between alcohol consumption and stroke because drinking patterns may be diverse even if the average amount or frequency of alcohol consumption is similar. Therefore, we combined these two drinking characteristics and further found that the risk of total stroke was reduced by 61% (95% CI: 0.17–0.89), in a drinking pattern of 6–7 days per week but less than 36 g/d, suggesting that a relatively healthy drinking pattern is equally important.

Previous studies have found a positive association between high alcohol consumption and stroke [5, 6, 15, 16], which differs from our findings. A possible reason for this discrepancy is the difference in the definition of alcohol consumption. Our study used a lower threshold of high alcohol consumption (> 36 g/d). For example, the Japan Public Health Center-based Prospective Study (JPHC Study) defined heavy drinkers as ≥ 450 g of ethanol per week [36]; another meta-analysis that included 35 observational studies defined high alcohol consumption as > 60 g of alcohol intake per day [6]. For further comparisons, we used an alternative categorization of alcohol consumption [37, 38], with a higher threshold (Supplementary Table 5). However, comparing the two alcohol consumption classifications, we found that heavy drinking (> 60 g/d or > 36 g/d) was not associated with stroke risk. This may be attributed to potential differential effects in the study populations.

In exploring the association between drinking characteristics and stroke in different gender populations, we found that alcohol consumption was strongly associated with a reduced risk of stroke in men. Still, such an association was not observed in women. Further, the significant interaction between gender and alcohol consumption and frequency of drinking (P_interaction_ was 0.009 and 0.028, respectively) also indicates that the association between alcohol consumption and stroke risk was more prominent in men, which is consistent with previous studies. For example, in a prospective cohort study of 21,860 men with an average follow-up of 21.6 years, the relative risk of total stroke with one drink per week was 0.80 (95% CI: 0.66–0.97) compared with men who drank less than one drink per week [39]. A physician health study found (with an average follow-up of 12.2 years) a 25% lower risk of total stroke in men who drank moderately (2–4 drinks per week) compared with participants who drank less than one drink per week [7]. And our study found that moderate alcohol consumption in men reduced the risk of stroke by 52% (95% CI: 0.25–0.92). While for example, a previous cohort study among 87,526 female nurses aged 34 to 59 years showed a relative risk of ischemic stroke of 0.3 (95% CI: 0.1–0.7) for 5 to 14 g of alcohol intake per day compared with non-drinkers [11]. A nurses’ health study also showed that light to moderate alcohol consumption in women was associated with a reduced risk of total stroke [9]. But this similar association was not observed in women in our study. Studies suggest that this gender difference may be due to differences in the metabolic and biological effects of alcohol and sensitivity to alcohol [40]. In addition, it may be because the participants who drank alcohol in our study were usually men, with 89.3% and 10.7% of drinkers being men and women, respectively, resulting in the association between alcohol consumption and stroke risk not being prominent in women.

Evidence suggests that the protective effect of alcohol consumption against stroke may be because moderate alcohol consumption is more conducive to improving the body’s lipid composition, significantly increasing blood high-density lipoprotein cholesterol (HDL) levels [41]. In addition, a randomized controlled trial also showed [42] that moderate alcohol consumption significantly increased circulating levels of total lipocalin and high molecular weight lipocalin compared to no alcohol consumption. Also, moderate alcohol consumption decreases platelet aggregation and fibrinogen concentration and reduces thrombosis, thereby reducing the risk of stroke [43].

Our study has several advantages. First, this study was conducted in Chongqing, China, a region with a highly spicy diet with an annual per capita consumption of 95.6 kg of chili peppers [44], where previous studies have shown that chili pepper consumption is associated with hypertension [45], obesity [46], and lipid disorders [47], and where residents often consume spicy food in conjunction with alcohol consumption. In addition, there was a significant interaction between spicy food intake and frequency of alcohol consumption (P_interaction_=0.017). Therefore, considering this dietary characteristic, we included spicy food intake when making adjustments for confounders. Second, our study was based on a large sample prospective cohort study design with more robust causal validation and long-term follow-up through reliable association to surveillance systems and supplemented with questionnaires. Finally, we comprehensively considered the effects of different drinking characteristics and patterns on stroke and stroke subtypes to evaluate the association between alcohol consumption and stroke risk.

This study also has some limitations. Firstly, baseline information on alcohol consumption was self-reported, which may lead to recall bias. Future studies should assess under-reporting of alcohol intake and possibly use new methods based on metabolic analysis to estimate alcohol consumption [48], which is expected to avoid errors in self-reporting of alcohol consumption. Secondly, although we strictly controlled for confounders, other potential residual confounders that could not be eliminated may have influenced our findings somewhat. Thirdly, We only collected information on alcohol use at baseline and did not investigate changes in alcohol use during follow-up. Therefore, alcohol consumption and other time-varying covariates may lead to residual confounding. Finally, our study was limited to Chongqing in southwest China and only represented some Chinese population. More prospective studies exploring the relationship between alcohol consumption and stroke risk in other regions are needed.

## Conclusion

In conclusion, moderate drinking (13–36 g/d) and drinking 6–7 days per week are associated with a reduced risk of total stroke. Studies on alcohol consumption should not only focus on the amount and frequency of alcohol consumption but also consider the drinking pattern. This study found that drinking 6–7 days per week but with an average of less than 36 g/d was a beneficial drinking pattern, and this drinking pattern may be associated with a lower risk of total stroke. Drinking alcohol is part of the lifestyle. Advice on alcohol consumption should be cautious, given the health risks it may pose to other diseases.

### Electronic supplementary material

Below is the link to the electronic supplementary material.

Supplementary Material 1

Supplementary Material 2

## Data Availability

The datasets generated and/or analyzed during the current study are not publicly available due the data contain sensitive information on participants but are available from the corresponding author upon reasonable request.

## Abbreviations

HR: Risk ratio
95% CI: 95% confidence interval
WHO: World Health Organization
BMI: Body mass index
FBG: Fasting Blood Glucose
SBP: Systolic blood pressure
DBP: Diastolic blood pressure
TC: Serum total cholesterol
TG: Triacylglycerol
LDL-C: Low-density lipoprotein cholesterol
HDL-C: High-density lipoprotein cholesterol
