# Supplementary material for: Association between alcohol consumption and risk of stroke among adults: results from a prospective cohort study in Chongqing, China
Source: BMC Public Health. 2023 Aug 22;23:1593. doi: 10.1186/s12889-023-16361-9 (PMC10464090; doi:10.1186/s12889-023-16361-9)
Supplement: Supplementary file 1 — Supplementary Material 1 [file 12889_2023_16361_MOESM1_ESM.docx]

| **Alcohol consumption** | | | | | | |
| --- | --- | --- | --- | --- | --- | --- |
| C1 | Have you had a drink in the last 24 hours? | | □ Yes  □ No | | | |
| C2 | How often have you had a drink in the past year? | | □ Never or hardly ever drinks alcohol ••••🡺  □ Only drink occasionally on special occasions, such as festivals or holidays  □ Drink only during certain months of the year (e.g., busy farming or summer) and not during other seasons  □ It is drunk every month of the year, regardless of season, but less than once a week  □ Drink alcohol at least once a week throughout the year, regardless of season ••••🡺 | | | D1  C4 |
|  | C2 Instructions for filling in the form: pay attention to distinguishing the time relationship between a season, a month, and a week. | | | | | |
| C3 | C3a Have you ever had a drink every week for at least a year? | | □ Yes ••••🡺  □ No ••••🡺 | | | C3b |
|  |  |  |  |  |  | D1 |
|  | C3b How long have you stopped doing this (Drinking every week for a year)? | | □□Year ••••🡺 | | | D1 |
|  | C3 Instructions for filling in the form: two details need to be taken into account here: 1. Last for at least a year; 2. Drink every week (at least once a week). | | | | | |
| C4 | On average, how many days per week have you drunk alcohol in the past year? | | □ 1-2d/week  □ 3-5d/week  □ 6-7d/week | | |  |
| C5 | About how old have you been since you started drinking alcohol every week? | | □□Years old | | |  |
| C6 | How much alcohol do you drink daily under three different conditions? (If you drink extra alcohol in extraordinary circumstances, you can select up to three alcohol simultaneously.) | | | | | |
|  | | Under general circumstances (choose one) | | When drinking a lot of alcohol due to particular circumstances (up to 3 choices) | Last time you had a drink (choose up to three) | |
| Beer | | □□bottle/week | | □□bottle/week | □□bottle/week | |
| Rice wine | | □□50g/week | | □□50g/day | □□50g/day | |
| Wine | | □□50g/week | | □□50g/day | □□50g/day | |
| High-alcohol liquor | | □□50g/week | | □□50g/day | □□50g/day | |
| Low-alcohol liquor | | □□50g/week | | □□50g/day | □□50g/day | |
| C6 Instructions for filling in the form: Each beer bottle shall be 500ml. | | | | | | |
