# Supplementary material for: Association between alcohol consumption and risk of stroke among adults: results from a prospective cohort study in Chongqing, China
Source: BMC Public Health. 2023 Aug 22;23:1593. doi: 10.1186/s12889-023-16361-9 (PMC10464090; doi:10.1186/s12889-023-16361-9)
Supplement: Supplementary file 2 — Supplementary Material 2 [file 12889_2023_16361_MOESM2_ESM.docx]

**Supplementary Table 1.** Association between alcohol consumption and stroke risk among 22,091 participants (HR and 95% CI).

| **Alcohol consumption (g/d)** | **Incidence Density**  **(1/100,000 Person-Years)** | **Model 1** | **Model 2** |
| --- | --- | --- | --- |
| **Total stroke (N=310)** |  |  |  |
| 0 | 375.55 | 1.00 | 1.00 |
| ＞0 to≤20 | 253.51 | 0.68 (0.39,1.18) | 0.56 (0.32,0.99) |
| ＞20 to≤40 | 8.33 | 0.64 (0.30,1.36) | 0.46 (0.21,0.99) |
| ＞40 to≤60 | 438.16 | 1.17 (0.58,2.36) | 0.72 (0.35,1.48) |
| ＞60 | 587.89 | 1.57 (0.81,3.05) | 0.69 (0.35,1.38) |
| **Ischemic stroke (N=245)** |  |  |  |
| 0 | 299.89 | 1.00 | 1.00 |
| ＞0 to≤20 | 195.01 | 0.65 (0.35,1.23) | 0.54 (0.28,1.03) |
| ＞20 to≤40 | 7.14 | 0.69 (0.31,1.55) | 0.51 (0.22,1.16) |
| ＞40 to≤60 | 328.62 | 1.10 (0.49,2.47) | 0.71 (0.31,1.62) |
| ＞60 | 326.60 | 1.09 (0.45,2.65) | 0.51 (0.20,1.25) |
| **Hemorrhagic stroke (N=59)** |  |  |  |
| 0 | 70.16 | 1.00 | 1.00 |
| ＞0 to≤20 | 58.50 | 0.83 (0.26,2.68) | 0.68 (0.21,2.23) |
| ＞20 to≤40 | — | — | — |
| ＞40 to≤60 | 109.54 | 1.56 (0.38,6.41) | 0.82 (0.19,3.53) |
| ＞60 | 195.96 | 2.79 (0.87,8.94) | 1.13 (0.33,3.83) |

Model 1: unadjusted crude model; Model 2: adjusted for gender, age, education level, marital status, smoking status, physical exercise, excessive intake of red meat, insufficient intake of vegetables and fruits, intake of spicy food, BMI, hypertension, diabetes, and dyslipidemia.

**Supplementary Table 2.** Association between different drinking characteristics and drinking patterns and stroke events in the male population (HR and 95% CI).

| **Drinking characteristics** | **Incidence Density**  **(1/100,000 Person-Years)** | **Model 1** | **Model 2** |
| --- | --- | --- | --- |
| **Total stroke**  **（N=171）** |  |  |  |
| Nondrinkers | 473.62 | 1.00 | 1.00 |
| Degree of alcohol intake |  |  |  |
| light | 344.51 | 0.36（0.73,1.44） | 0.71（0.36,1.41） |
| moderate | 235.07 | **0.50（0.26,0.95）** | **0.48（0.25,0.92）** |
| high | 455.14 | 0.97（0.57,1.64） | 0.65（0.37,1.12） |
| Drinking frequency |  |  |  |
| 1-2d/week | 209.82 | 0.45（0.20,1.01） | 0.61（0.27,1.40） |
| 3-5d/week | 282.27 | 0.60（0.29,1.22） | 0.65（0.32,1.34） |
| 6-7d/week | 447.59 | 0.95（0.59,1.52） | **0.58（0.36,0.94）** |
| **Ischemic stroke**  **（N=129）** |  |  |  |
| Nondrinkers | 356.08 | 1.00 | 1.00 |
| Degree of alcohol intake |  |  |  |
| light | 267.95 | 0.76（0.35,1.63） | 0.70（0.33,1.52） |
| moderate | 211.57 | 0.60（0.30,1.18） | 0.56（0.28,1.12） |
| high | 303.42 | 0.86（0.45,1.64） | 0.57（0.29,1.10） |
| Drinking frequency |  |  |  |
| 1-2d/week | 174.85 | 0.49（0.20,1.21） | 0.65（0.26,1.61） |
| 3-5d/week | 246.99 | 0.70（0.32,1.50） | 0.74（0.34,1.61） |
| 6-7d/week | 313.31 | 0.88（0.51,1.54） | **0.53（0.30,0.94）** |
| **Hemorrhagic stroke（N=38）** |  |  |  |
| Nondrinkers | 110.63 | 1.00 | 1.00 |
| Degree of alcohol intake |  |  |  |
| light | 76.56 | 0.70（0.17,2.91） | 0.82（0.20,3.48） |
| moderate | — | — | — |
| high | 121.37 | 1.10（0.39,3.12） | 0.77（0.26,2.27） |
| Drinking frequency |  |  |  |
| 1-2d/week | 34.97 | 0.32（0.04,3.33） | 0.51（0.07,3.82） |
| 3-5d/week | 35.28 | 0.32（0.04,2.35） | 0.40（0.05,2.94） |
| 6-7d/week | 89.52 | 0.81（0.29,2.30） | 0.53（0.18,1.56） |

Model 1: unadjusted crude model; Model 2: adjusted for age, education level, marital status, smoking status, physical exercise, excessive intake of red meat, insufficient intake of vegetables and fruits, intake of spicy food, BMI, hypertension, diabetes, and dyslipidemia.

**Supplementary Table 3.** Association between different drinking patterns and stroke events in the male population (HR and 95% CI).

| **Drinking pattern** | | **Incidence Density**  **(1/100,000 Person-Years)** | **Model 1** | **Model 2** |
| --- | --- | --- | --- | --- |
| **Total stroke**  **（N=171）** |  |  |  |  |
| Nondrinkers |  | 473.62 | 1.00 | 1.00 |
| 1-2d/week | 0-36g/d | 227.28 | 0.48（0.21,1.09） | 0.66（0.29,1.50） |
|  | ＞36g/d | — | — | — |
| 3-5d/week | 0-36g/d | 335.83 | 0.71（0.33,1.52） | 0.74（0.34,1.60） |
|  | ＞36g/d | 133.37 | 0.28（0.04,2.03） | 0.36（0.05,2.56） |
| 6-7d/week | 0-36g/d | 280.09 | 0.59（0.26,1.35） | **0.41（0.18,0.93）** |
|  | ＞36g/d | 601.82 | 1.28（0.74,2.21） | 0.74（0.42,1.31） |
| **Ischemic stroke（N=129）** |  |  |  |  |
| Nondrinkers |  | 356.08 | 1.00 | 1.00 |
| 1-2d/week | 0-36g/d | 189.40 | 0.54（0.22,1.31） | 0.70（0.28,1.74） |
|  | ＞36g/d | — | — | — |
| 3-5d/week | 0-36g/d | 287.86 | 0.81（0.36,1.85） | 0.84（0.36,1.92） |
|  | ＞36g/d | 133.37 | 0.38（0.05,2.70） | 0.46（0.06,3.34） |
| 6-7d/week | 0-36g/d | 46.68 | 0.66（0.27,1.61） | 0.43（0.18,1.07） |
|  | ＞36g/d | 386.89 | 1.09（0.55,2.15） | 0.64（0.32,1.29） |
| **Hemorrhagic stroke（N=38）** |  |  |  |  |
| Nondrinkers |  | 110.63 | 1.00 | 1.00 |
| 1-2d/week | 0-36g/d | 37.88 | 0.34（0.05,2.52） | 0.56（0.08,4.21） |
|  | ＞36g/d | — | — | — |
| 3-5d/week | 0-36g/d | 47.98 | 0.44（0.06,3.19） | 0.52（0.07,3.86） |
|  | ＞36g/d | — | — | — |
| 6-7d/week | 0-36g/d | — | — | — |
|  | ＞36g/d | 171.95 | 1.56（0.55,4.41） | 0.89（0.30,2.63） |

Model 1: unadjusted crude model; Model 2: adjusted for age, education level, marital status, smoking status, physical exercise, excessive intake of red meat, insufficient intake of vegetables and fruits, intake of spicy food, BMI, hypertension, diabetes, and dyslipidemia.

**Supplementary Table 4.** Association between drinking characteristics and stroke events among 18,205 participants (HR and 95% CI).

| **Drinking characteristics** | **Incidence Density**  **(1/100,000 Person-Years)** | **Model 1** | **Model 2** |
| --- | --- | --- | --- |
| **Total stroke**  **（N=216）** |  |  |  |
| Nondrinkers | 312.39 | 1.00 | 1.00 |
| Degree of alcohol intake |  |  |  |
| light | 251.62 | 0.81（0.38,1.72） | 0.59（0.26,1.34） |
| moderate | 186.13 | 0.60（0.28,1.27） | **0.39（0.18,0.85）** |
| high | 528.79 | 1.70（0.99,2.92） | 0.62（0.33,1.14） |
| Drinking frequency |  |  |  |
| 1-2d/week | 144.31 | 0.46（0.17,1.25） | 0.45（0.16,1.26） |
| 3-5d/week | 278.50 | 0.89（0.42,1.90） | 0.65（0.30,1.42） |
| 6-7d/week | 435.33 | 1.40（0.85,2.29） | **0.50（0.29,0.88）** |
| **Ischemic stroke**  **（N=161）** |  |  |  |
| Nondrinkers | 237.62 | 1.00 | 1.00 |
| Degree of alcohol intake |  |  |  |
| light | 143.78 | 0.61（0.23,1.64） | 0.49（0.18,1.35） |
| moderate | 159.54 | 0.67（0.30,1.52） | 0.44（0.19,1.01） |
| high | 302.17 | 1.27（0.63,2.60） | 0.47（0.21,1.03） |
| Drinking frequency |  |  |  |
| 1-2d/week | 108.23 | 0.46（0.15,1.43） | 0.48（0.15,1.52） |
| 3-5d/week | 238.72 | 1.01（0.44,2.28） | 0.75（0.32,1.74） |
| 6-7d/week | 230.47 | 0.97（0.50,1.91） | **0.36（0.17,0.74）** |
| **Hemorrhagic stroke**  **（N=50）** |  |  |  |
| Nondrinkers | 69.79 | 1.00 | 1.00 |
| Degree of alcohol intake |  |  |  |
| light | 107.84 | 1.55（0.48,5.01） | 0.98（0.23,4.18） |
| moderate | — | — | — |
| high | 188.85 | **2.71（1.07,6.84）** | 0.99（0.33,2.99） |
| Drinking frequency |  |  |  |
| 1-2d/week | 36.08 | 0.52（0.07,3.77） | 0.41（0.05,3.49） |
| 3-5d/week | 39.79 | 0.57（0.08,4.15） | 0.37（0.05,2.90） |
| 6-7d/week | 153.65 | 2.20（0.94,5.18） | 0.74（0.27,2.07） |

Model 1: unadjusted crude model; Model 2: adjusted for gender, age, education level, marital status, smoking status, physical exercise, excessive intake of red meat, insufficient intake of vegetables and fruits, intake of spicy food, BMI, hypertension, diabetes, dyslipidemia, and drinking quantity*gender.

**Supplementary Table 5.** Association between drinking patterns and stroke events among 18,205 participants (HR and 95% CI).

| **Drinking pattern** | | **Incidence Density**  **(1/100,000 Person-Years)** | **Model 1** | **Model 2** |
| --- | --- | --- | --- | --- |
| **Total stroke**  **（N=216）** |  |  |  |  |
| Nondrinkers |  |  | 1.00 | 1.00 |
| 1-2d/week | 0-36g/d | 154.40 | 0.50（0.18,1.33） | 0.54（0.20,1.48） |
|  | ＞36g/d | — | — | — |
| 3-5d/week | 0-36g/d | 311.23 | 1.00（0.44,2.25） | 0.74（0.32,1.69） |
|  | ＞36g/d | 170.77 | 0.55（0.08,3.91） | 0.47（0.06,3.38） |
| 6-7d/week | 0-36g/d | 197.59 | 0.63（0.24,1.71） | **0.24（0.08,0.76）** |
|  | ＞36g/d | 691.23 | **2.22（1.26,3.89）** | 0.68（0.36,1.29） |
| **Ischemic stroke（N=161）** |  |  |  |  |
| Nondrinkers |  |  | 1.00 | 1.00 |
| 1-2d/week | 0-36g/d | 115.80 | 0.49（0.16,1.53） | 0.52（0.16,1.67） |
|  | ＞36g/d | — | — | — |
| 3-5d/week | 0-36g/d | 259.36 | 1.09（0.45,2.67） | 0.80（0.32,1.99） |
|  | ＞36g/d | 170.77 | 0.72（0.10,5.15） | 0.62（0.09,4.50） |
| 6-7d/week | 0-36g/d | 98.80 | 0.42（0.10,1.68） | **0.21（0.05,0.84）** |
|  | ＞36g/d | 372.20 | 1.57（0.74,3.35） | 0.49（0.21,1.13） |
| **Hemorrhagic stroke**  **（N=50）** |  |  |  |  |
| Nondrinkers |  |  | 1.00 | 1.00 |
| 1-2d/week | 0-36g/d | 38.60 | 0.56（0.08,4.03） | 0.67（0.09,5.05） |
|  | ＞36g/d | — | — | — |
| 3-5d/week | 0-36g/d | 51.87 | 0.75（0.10,5.41） | 0.58（0.08,4.36） |
|  | ＞36g/d | — | — | — |
| 6-7d/week | 0-36g/d | 49.40 | 0.71（0.10,5.16） | — |
|  | ＞36g/d | 265.86 | **3.80（1.50,9.61）** | 1.15（0.38,3.51） |

Model 1: unadjusted crude model; Model 2: adjusted for gender, age, education level, marital status, smoking status, physical exercise, excessive intake of red meat, insufficient intake of vegetables and fruits, intake of spicy food, BMI, hypertension, diabetes, dyslipidemia, and drinking quantity*gender.


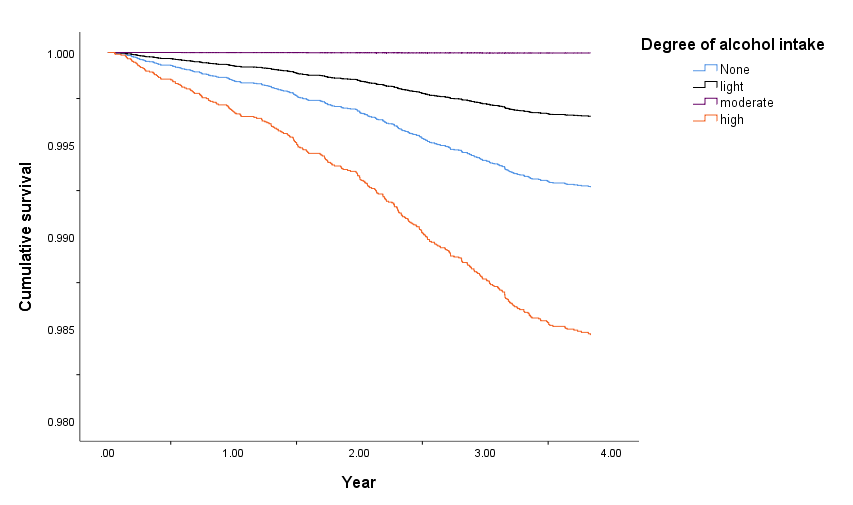


**Supplementary FIGURE 1.** Survival curves for different degrees of alcohol consumption. The survival curves were controlled by gender, age, education level, marital status, smoking status, physical exercise, excessive intake of red meat, insufficient intake of vegetables and fruits, intake of spicy food, BMI, hypertension, diabetes, dyslipidemia, and drinking quantity*gender. Log-rank test＜0.05.


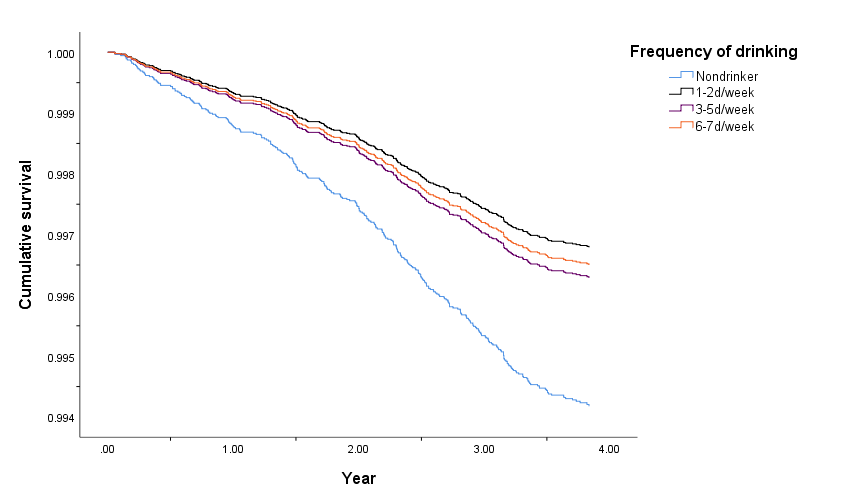


**Supplementary FIGURE 2.** Survival curves for different frequencies of alcohol consumption. The survival curves were controlled by gender, age, education level, marital status, smoking status, physical exercise, excessive intake of red meat, insufficient intake of vegetables and fruits, intake of spicy food, BMI, hypertension, diabetes, dyslipidemia, and drinking quantity*gender. Log-rank test＜0.05.
